# Supplementary material for: The genome sequence of a snipefly, Rhagio lineola Fabricius, 1794
Source: Wellcome Open Res. 2025 Mar 19;10:154. [Version 1] doi: 10.12688/wellcomeopenres.23901.1 (PMC12501595; doi:10.12688/wellcomeopenres.23901.1)
Supplement: Peer review history

# Reviewer report: Reviewer response for version 1

**DOI:** 10.21956/wellcomeopenres.26368.r132641

**Published:** 2025-10-06

**Authors:** Rodolpho S. T. Menezes

**Affiliations:** 1 State University of Santa Cruz, Ilhéus, Brazil

The data note by Sivell et al. presents a genome assembly from an individual male of the snipefly *Rhagio lineola *Fabricius, 1794. The final assembly is 1,654.96 Mb. They also assembled the mitogenome (16.64 kb).

 This genome information will be interesting for comparative genomics investigation and also for evolution, and systematics studies. The methodology is thorough and explained and the figures are well constructed and the data note is well written. I have no notes to add.

Are sufficient details of methods and materials provided to allow replication by others?

Yes

Is the rationale for creating the dataset(s) clearly described?

Yes

Are the datasets clearly presented in a useable and accessible format?

Yes

Are the protocols appropriate and is the work technically sound?

Yes

Reviewer Expertise:

Phylogenomics, Cytogenetics, Molecular Phylogenetics.

I confirm that I have read this submission and believe that I have an appropriate level of expertise to confirm that it is of an acceptable scientific standard.

# Reviewer report: Reviewer response for version 1

**DOI:** 10.21956/wellcomeopenres.26368.r121734

**Published:** 2025-05-08

**Authors:** Carlos Congrains

**Affiliations:** 1 U.S. Department of Agriculture-Agricultural Research Service, Hilo, Hawaii, USA

The authors generated a genome assembly for a male of the small insect *Rhagio lineola*, which is native to the Palearctic region and introduced to the Nearctic as part of the Darwin Tree of Life Project. The assembly produced based on PacBio Hi-Fi and chromatin conformation capture (Hi-C) data is highly contiguous and complete in terms of gene content. Based on coverage analysis, the authors identified four autosomal chromosomes and the X chromosome, but not the Y chromosome. They also assembled and annotated the mitochondrial genome. The authors demonstrated that this genome assembly meets the criteria for a high-quality genome regarding contiguity, gene content, quality value, and the absence of detectable contaminants.

 The authors generated a genome assembly for a male of the small insect *Rhagio lineola*, which is native to the Palearctic region and introduced to the Nearctic as part of the Darwin Tree of Life Project. The assembly produced based on PacBio Hi-Fi and chromatin conformation capture (Hi-C) data is highly contiguous and complete in terms of gene content. Based on coverage analysis, the authors identified four autosomal chromosomes and the X chromosome, but not the Y chromosome. They also assembled and annotated the mitochondrial genome. The authors demonstrated that this genome assembly meets the criteria for a high-quality genome regarding contiguity, gene content, quality value, and the absence of detectable contaminants.

Are sufficient details of methods and materials provided to allow replication by others?

Yes

Is the rationale for creating the dataset(s) clearly described?

Yes

Are the datasets clearly presented in a useable and accessible format?

Yes

Are the protocols appropriate and is the work technically sound?

Yes

Reviewer Expertise:

Evolutionary genomics

I confirm that I have read this submission and believe that I have an appropriate level of expertise to confirm that it is of an acceptable scientific standard.
